# Supplementary material for: In Vitro Digestion and Colonic Fermentation of UHT Treated Faba Protein Emulsions: Effects of Enzymatic Hydrolysis and Thermal Processing on Proteins and Phenolics
Source: Nutrients. 2022 Dec 24;15(1):89. doi: 10.3390/nu15010089 (PMC9824445; doi:10.3390/nu15010089)
Supplement: Supplementary file 1 [file nutrients-15-00089-s001.zip › nutrients-2061017-supplementary.pdf]

Supplementary file

Table S1. The significant difference analysis result of Total SCFAs.

Samples	HA_0	HA_5	HA_{10}	HA_{30}	EA_0	EA_5	EA_{10}	EA_{30}
0h	1.68 ± 0.01^c	1.76 ± 0.01^b	1.26 ± 0.01^f	1.54 ± 0.01^d	1.79 ± 0.01^b	1.86 ± 0.01^a	1.40 ± 0.01^e	1.84 ± 0.01^a
2h	3.23 ± 0.01^c	3.68 ± 0.01^a	2.99 ± 0.01^e	3.67 ± 0.01^a	2.73 ± 0.01^f	3.06 ± 0.01^d	3.32 ± 0.01^b	3.30 ± 0.01^b
4h	2.66 ± 0.01^e	4.62 ± 0.01^a	3.26 ± 0.01^c	3.14 ± 0.01^d	2.45 ± 0.01^f	0.13 ± 0.01^h	1.31 ± 0.01^g	3.45 ± 0.01^b
8h	11.59 ± 0.01^b	11.60 ± 0.01^b	15.35 ± 0.01^a	8.65 ± 0.01^f	9.88 ± 0.01^c	9.40 ± 0.01^d	9.04 ± 0.01^e	8.01 ± 0.01^g
16h	13.72 ± 0.01^g	14.79 ± 0.01^f	18.70 ± 0.01^d	25.88 ± 0.01^a	20.27 ± 0.01^b	19.30 ± 0.01^c	17.70 ± 0.01^e	13.18 ± 0.01^h
24h	13.01 ± 0.01^f	13.73 ± 0.01^f	17.58 ± 0.01^b	20.13 ± 0.01^a	16.81 ± 0.01^c	16.00 ± 0.01^d	14.81 ± 0.01^e	11.46 ± 0.01^g

The significant difference analysis result of Total SCFAs has been updated in the supplementary file as an example.
